# Queries Respecting the Origin of Azote in Herbivorous Animals

**Published:** 1819-08

**Authors:** 


FOR THE LONDON MEDICAL AND PHYSICAL JOURNAL.

### *Queries respecting the Origin of Azote in Herbivorous Animals.* By SILVIUS.

WISHING for accurate information on the following subjects, your Journal appeared to be the medium through which I might probably obtain it: by giving, therefore, a place in it to the following queries, you will confer an obligation on your constant reader, SILVIUS.

*London; June 6th,* 1819.

Has it been proved by decisive experiments, that azote is not absorbed from the atmosphere by the skin?

Is it not determined that absorption of azote is not effected by the lungs?

If the first query can be replied to in the affirmative, where is an account of those experiments to be found?

Does azote exist in as great proportion in the chyle of *herbivorous* as in the same fluid of *carnivorous* animals; or is the azote found in the chyle of herbivorous animals, only derived from the secretions poured into the alimentary canal during digestion?

Is the hypothesis of a new combination of the elements of vegetable substances in the alimentary canal, that most generally admitted in Europe as explanatory of the mystery of the origin of azote in herbivorous animals?

What do the best chemists, who still call azote an *element*, say to the preceding hypothesis?

